# Nuclear-Cytoplasmic Conflict in Pea (*Pisum sativum* L.) Is Associated with Nuclear and Plastidic Candidate Genes Encoding Acetyl-CoA Carboxylase Subunits

**DOI:** 10.1371/journal.pone.0119835

**Published:** 2015-03-19

**Authors:** Vera S. Bogdanova, Olga O. Zaytseva, Anatoliy V. Mglinets, Natalia V. Shatskaya, Oleg E. Kosterin, Gennadiy V. Vasiliev

**Affiliations:** 1 Institute of Cytology and Genetics of Siberian Division of Russian Academy of Sciences, Novosibirsk, Russia; 2 Novosibirsk State University, Novosibirsk, Russia; Saint Mary's University, CANADA

## Abstract

In crosses of wild and cultivated peas (*Pisum sativum* L.), nuclear-cytoplasmic incompatibility frequently occurs manifested as decreased pollen fertility, male gametophyte lethality, sporophyte lethality. High-throughput sequencing of plastid genomes of one cultivated and four wild pea accessions differing in cross-compatibility was performed. Candidate genes for involvement in the nuclear-plastid conflict were searched in the reconstructed plastid genomes. In the annotated *Medicago truncatula* genome, nuclear candidate genes were searched in the portion syntenic to the pea chromosome region known to harbor a locus involved in the conflict. In the plastid genomes, a substantial variability of the *accD* locus represented by nucleotide substitutions and indels was found to correspond to the pattern of cross-compatibility among the accessions analyzed. Amino acid substitutions in the polypeptides encoded by the alleles of a nuclear locus, designated as *Bccp3*, with a complementary function to *accD*, fitted the compatibility pattern. The *accD* locus in the plastid genome encoding beta subunit of the carboxyltransferase of acetyl-coA carboxylase and the nuclear locus *Bccp3* encoding biotin carboxyl carrier protein of the same multi-subunit enzyme were nominated as candidate genes for main contribution to nuclear-cytoplasmic incompatibility in peas. Existence of another nuclear locus involved in the *accD*-mediated conflict is hypothesized.

## Introduction

Hybridization of plant species and subspecies is an important process both for natural evolution [1] and selection for agriculture. For the establishment of new forms the restriction of gene flow via prezygotic or postzygotic crossing barriers is important. Although in general prezygotic isolation more readily imposes reproductive barriers in evolution, in some plants postzygotic isolation plays a substantial role [2]. Three types of postzygotic reproductive barriers were genetically studied, cytonuclear incompatibility, hybrid sterility and hybrid breakdown [3].

Breeding programs for crop improvement [4] often employ wide hybridization of plant cultivars with their wild relatives. However, such hybridization is often hindered by reproductive barriers frequently manifested as hybrid sterility [5]. During recent years, a substantial success has been achieved in understanding the genetic basis underlying hybrid incompatibilities. In rice (*Oryza sativa*), several nuclear loci responsible for incompatibility of two cultivated subspecies *O*. *s*. subsp. *indica* and *O*. *s*. subsp. *japonica* were cloned and characterized [6–9].

Hybrid incompatibility in plants often results from nuclear-organellar conflict according to the Bateson-Dobzhansky-Muller model [10]. In the course of evolution, regulatory pathways are established between the nuclear, plastid and mitochondrial genomes, so that different cellular genomes become functionally coadapted. The established interaction may be disrupted if alien genomes are brought together by hybridization. The more divergent are organellar genomes the higher is the probability of disharmonic relation between them and nuclear genome in inter-species hybrids and the more severe are postzygotic barriers for the gene flow [11]. One of the well-known manifestations of the nuclear-mitochondrial conflict is cytoplasmic male sterility (CMS) which frequently occurs in plant hybrids and consists in decreased male fertility [12]. At the molecular level, CMS commonly results from chimeric mitochondrial DNA transcripts containing essential genes. The negative effect of such aberrant transcripts is usually compensated by nuclear genes encoding RNA-binding proteins [13].

Some cases were molecularly studied where hybridization affected the interaction between nuclear and plastid genes. Nuclear-cytoplasmic conflict has been described in somatic hybrids (cybrids) obtained by protoplast fusion in Solanaceae plants [14, 15]. An analysis of nightshade/tobacco hybrids showed that absence of a gene responsible for editing of plastid ATPase α-subunit mRNA in the nightshade nuclear genome resulted in disruption of tobacco chloroplast function [16]. Among species of the genus *Oenothera*, a conflict of the plastid and nuclear genomes has been known for a long time [17]. Recently, based on the whole genome sequencing of chloroplast DNA in five *Oenothera* species [18], the promoter region between *psbB* and *clpP* was suggested among other candidates to contribute to the plastome-genome incompatibility [19]. Nuclear counterparts in the studied nuclear-plastid incompatibilities remained obscure.

In the pea (*Pisum sativum* L.), we described nuclear-plastid incompatibility in crosses of wild representatives with cultivated forms [20, 21] and genetically mapped two complementary unlinked nuclear genes, *Scs1* and *Scs2* involved in the conflict [22]. In crosses of cultivated peas with the wild accession VIR320 as donor of cytoplasm, heterozygotes for both *Scs1* and *Scs2* were very weak with mosaic chlorophyll deficiency, reduction of blade organs, low pollen and seed fertility, poorly developed roots. Heterozygotes for either of the genes *Scs1* or *Scs2* had slightly reduced chlorophyll pigmentation and good seed production, while the pollen fertility was decreased to about 50–70% [23]. We further focused on the more detailed genetic analysis of the *Scs1* locus since its manifestation was more pronounced. Unlike *Scs2*, *Scs1* allele from the cultivated parent was shown to be both sporophyte and male gametophyte lethal in the background of the alien cytoplasm. It was genetically mapped on Linkage Group (LG) III [22].

In the present work we sequenced entire plastid genomes in a set of pea accessions known to be compatible or incompatible with the nuclear *Scs1* of the cultivated tester line WL1238 [24]. We report on the plastid *accD* gene coding for the acetyl-CoA carboxylase beta subunit and a nuclear gene *Bccp3* coding for the biotin carboxyl carrier protein of acetyl-CoA carboxylase as an ideal pair of candidates for the role of the incompatibility genes in question.

## Materials and Methods

### Plant material

The common cultivated pea subspecies, *P*. *sativum* subsp. *sativum* L., was represented by a testerline WL1238, compatible with other germplasm of this subspecies.

Wild peas, presently grouped into the paraphyletic subspecies *P*. *s*. subsp. *elatius* (Bieb.) Schmahl. [25], were represented by the following accessions, compatible or incompatible with nuclear *Scs1* of WL1238. A plastid genome was considered to be incompatible with a nuclear *Scs1* allele if homozygotes for the paternal allele of a molecular marker closely linked to *Scs1* were absent or severely underrepresented in the F2 segregation [24]. Compatibility of the plastid genomes with the *Scs2* locus was not genetically studied, hence, the allelic state of this locus remained unknown.

The following accessions were used:

JI1794 (Israel, Holan Heights), plastid genome compatible with WL1238;
721 (Israel, Mt. Carmel; of the lines used by Ben-Ze’ev and Zohary [26]), plastid genome compatible with WL1238;
VIR320 (“Palestine”), plastid genome incompatible with WL1238;
L100 (Israel, Be’er Sheva; = 712 of Ben-Ze’ev and Zohary [26]; = JI3273; = PI560069), plastid genome incompatible with WL1238.
A detailed description of these accessions is available in [27, supplementary material].

### Plastid DNA extraction

Plastid DNA was extracted according to [28], with modifications. About 100–200 mg of fresh leaves from seedlings of each accession were rubbed through stainless steel tea-strainer (1x1 mm^2^) into 30 ml of cold chloroplast isolation buffer (0.33 M sorbitol, 0,1% BSA; 50 mM HEPES). Suspension was centrifuged at 4°C for 20 min at 335g. Supernatant was transferred to a fresh tube and gently underlayered with 5 ml of 60% sucrose, then the tube was centrifuged for 30 min at 4°C at 3300g. After centrifugation, the chloroplasts concentrated just above the sucrose layer. This zone was carefully transferred to a fresh tube, diluted with 30 ml of chloroplast isolation buffer and centrifuged at 3300g for 25 min at 4°C. The supernatant was discarded. To separate chloroplasts from mitochondria the pellet was resuspended in 6 ml of chloroplast isolation buffer and gently loaded onto a step gradient consisting of 6 ml of 60% sucrose, overlayed with 20 ml of 40% sucrose, then the tube was centrifuged for 17 min at 4°C at 20000 g.

The chloroplast band was collected and concentrated by centrifugation at 2000 g in microcentrifuge. The part of supernatant which did not contain chloroplasts was discarded. 100 μl of the remaining chloroplast suspension were gently mixed with 300 μl of CTAB lysis buffer (50 mM TrisHCl pH 8.0, 33 mM EDTA:Tris pH 7.0, 2M NaCl, 26 mM β-mercaptoethanol, 2% polyvinylpyrrolidone-10, 2.6% CTAB) and shaken on the rotator platform for one hour at 65°C at 5 sec/rev. For DNA extraction, 400 μl of chloroform were added and the tube was gently stirred, then centrifuged at 14000g in microcentrifuge. Upper layer was transferred to a fresh tube, mixed with 1,100 μl of dilution buffer (50mM TrisCl pH 8.0, 33mM EDTA:Tris pH 7.0, 1% CTAB), incubated overnight at 4°C and centrifuged at 21000g for 30 min. Supernatant was discarded, the pellet was resuspended in 200 μl of the buffer (10 mM TrisCl pH 8.0; EDTA:Tris pH 7.0, 1,5M NaCl) with 1 μl of RNAse A and shaken on the rotator platform at least 1 hour at 42°C until the pellet dissolved completely. One volume (200 μl) of chloroform was added for extraction, the tube was gently stirred and spun down in microcenrtifuge. The aqueous upper phase was transferred to a fresh tube, DNA was recovered by isopropanol precipitation. The pellet was washed by 80% ethanol, air-dried and dissolved in 100 μl of low-TE (10 mM Tris-HCl, 0.1 mM EDTA).

### Ion Torrent PGM sequencing

Plastid DNA was sequenced using the Ion Torrent platform. Libraries were prepared using the Ion Xpress Plus Fragment Library Kit (Life Technologies, USA) with enzymatic fragmentation following the kit protocol with 100 ng of input DNA. Libraries were constructed using the following adapters without barcoding, A: 5’-CCATCTCATCCCTGCGTGTCTCCGACTCAG; P1: 5’-CCTCTCTATGGGCAGTCGGTGAT. Library fraction 310–360 bp (median insert size ~260 bp) after adapter ligation was extracted using LabChip XT (Perkin-Elmer, USA), DNA 300 chips. Prior to amplification, the eluates from LabChip were concentrated and purified with 1.5 V of Agencourt AMPure XP beads, after 9 cycles of amplification libraries were purified using 1.5 V of Agencourt AMPure XP beads. The purity and concentration of libraries were analyzed by Bioanalyzer 2100 with DNA High Sensitivity Chip. Templates for Ion Torrent sequencing were prepared by using Ion PGM Template OT2 200 Kit. Libraries were sequenced on an Ion PGM using Ion316 chips deposited at full density following the protocol for 200 bp sequencing supplied by the manufacturer.

Representation of the plastid DNA in the sequenced sample was estimated using the BLAST utility [29] at NCBI (blast.ncbi.nlm.nih.gov). *Pisum sativum* chloroplast, complete genome (NC_014057) and *Vicia faba* mitochondrion, complete genome (KC189947) were used as a query against 10 random samples of about 10,000 reads filtered to get rid of reads shorter than 30 bases (115,627 in total) and the number of targets producing blast hits at the expectation threshold level of 10 was averaged over the 10 samples used to give an estimate of 77.29±0.41% of chloroplast DNA and 9.41±0.30% of mitochondrial DNA. The rest approx. 13.3% of reads were assumed to be represented by nuclear DNA.

### Plastid genome assembly

Plastid genomes were assembled starting with FASTQ format files using the MIRA4 program [30] with the default settings and ‘mapping’ option, with NC_014057 (*Pisum sativum* complete chloroplast genome) as the reference sequence. Assembly with the ‘de novo’ option provided contiguous sequence stretches used to resolve cases of deletions/insertions. The resulting assembly in the ACE format was visualized with the Tablet software [31] and checked manually. The evident discrepancies due to deletion/duplication (not nucleotide substitutions) between the analyzed and reference sequences were corrected using the data from ‘de novo’ contigs and the corrected version was used as a new reference for the second iteration of assembly with MIRA. This step was repeated 3–4 times, if necessary. In case of doubtful length of homopolymer stretches, typical for the Ion Torrent technology [32], their length was adjusted to fit the reference genome. Few gaps in the assemblies, confined to non-coding regions, were not filled and not analyzed.

### RNA isolation, cDNA synthesis and sequencing

Total RNA was extracted from young pea leaves with the use of SV Total RNA Isolation System, Promega (USA) according to manufacturer's instructions. cDNA synthesis was performed with the use of QT primer [33] and M-MLV reverse transcriptase Promega (USA) according to manufacturer's recommendations. Two forward primers, Ps_bccp-F2 5'-CTAATGAAAGTGGCGGAAATC, Ps_bccp-F3 5'-CGAAGCATTGGAGCAACAAAC, and two reverse primers, Ps_bccp-R2 5'-CATTCACTAAGACGCGTAATAAGG, Ps_bccp-R3 5'-TTCTGGAGATGATGTTGGTGG matching the GAMJ01025269 accession were used in all pairwise combinations to amplify cDNA fragments with the following PCR conditions: initial denaturation at 95°C for 3 min followed by 38 cycles of 30 s at 94°C, 30 s at 58°C, 1 min at 72°C, and final extension for 5 min at 72°C. PCR products were analyzed on 1.5% agarose gel in TAE buffer. If necessary, PCR-products were reamplified using 2 μl of 1:10 diluted PCR reaction as a template. Sequencing of the PCR-amplified products was performed with the use of BigDye terminators 3.1 and the same primers as for PCR at the SB RAS Genomics Core Facility.

### CAPS marker analysis

Locus genotyping was made via CAPS (Cleaved Amplified Polymorphic Sequences) approach [34]. Genomic DNA available from earlier experiments was PCR-amplified with the use of primers Ps_bccp-F4 (5'-GAGACTGAAATCGCTGAACTG) and Ps_bccp-R4 (5'-GTATGTATTGATACCAGAAGCC) designed to fit the cDNA sequence obtained in the present study. The following conditions were applied: initial denaturation at 95° for 3 min followed by 38 cycles of 30 s at 94°, 30 s at 58°, 1 min at 72°, and final extension for 5 min at 72°C. Five microliters of the PCR-reaction were digested with *Fsp*4HI endonuclease (Sibenzyme, Novosibirsk) according to the manufacturer's instructions and electrophoretically analyzed on 1.5% agarose gel in TAE. The allele inherited from VIR320 contained the recognition site, while the allele inherited from WL1238 did not.

### Genetic mapping

To genetically map the candidate locus *Bccp3*, the mapping RIL population was used which was obtained as F6 of the cross WL1238 x VIR320 and used earlier for the genetic analysis of the *Scs1* and *Scs2* loci [23]. The obtained data on the allelic state of the candidate locus together with the available data on the allelic state of LGIII markers were analyzed with the Mapmaker 3.0 software [35].

### Sequence data

The DNA sequences obtained in this study were submitted to the European Nucleotide Archive (ENA). These are reconstructed plastid genomes of five pea accessions (accession numbers HG966672-HG966676) and cDNA sequences from the *Bccp3* locus (accession numbers LK056919-LK056921, LK056923, LK056924).

## Results

### Plastid genome size

The plastid genome assemblies were circular molecules of the following sizes. WL1238–122,180 bp; VIR320–121,824 bp, of which 258 + 37 bp were two gaps in the assembly; L100–121,799 bp, of which 780 bp were 11 gaps of 17, 396, 10, 14, 5, 90, 84, 19, 26, 20, 99 bp in the assembly; 721–121,773 bp, of which 64 + 30 bp were two gaps in the assembly; JI1794–121,760 bp. All gaps were confined to non-coding regions, their length was assumed to be equal to that of the corresponding region of the WL1238 genome. The length of the reference genome NC_014057 (cultivar Feltham First) was 122,169 bp. Thus, plastid genomes of the wild peas generally tended to be shorter for about 400 bp.

All plastid genomes sequenced contained a deletion of 54 bp and insertion of 45 bp in the coding sequence of the *psaA* locus as compared to the reference genome of the Feltham First cultivar.

The plastid genome of JI1794 contained a 3442 bp inversion with one breakpoint in the intergenic spacer *psaI-accD* and the other in the intergenic spacer *psbI-trnS*. This inversion was predicted by Palmer et al. [36] based on restrictase analysis. General comparison of the reconstructed plastid genomes will be presented elsewhere.

### Search for plastid candidate loci involved in nuclear-cytoplasmic incompatibility

The reconstructed plastid genome of WL1238 was compared to those of the other four accessions, two of which had the cytoplasms incompatible with the nuclear *Scs1* of WL1238, namely, VIR320, L100, and two accessions, 721, JI1794, had the compatible cytoplasms [24]. The main guideline was that in the two incompatible plastid genomes, the candidate loci should differ from their orthologs in WL1238 and accessions compatible to it. List of all registered differences in the plastid genomes reconstructed is given in S1 Dataset. We detected 37 nucleotide substitutions and indels present in both VIR320 and L100 and absent in both 721 and JI1794. They were confined to 14 non-coding regions (intergenic spacers and introns) and 7 protein-coding loci. Theoretically, each of them could be considered as a candidate. However, to start with, we assumed (i) protein-coding loci to be more probable candidates and (ii) a difference in the protein structure to be a most likely cause of the nuclear-plastid incompatibility. Therefore, we excluded the non-coding regions and dismissed 5 synonymous nucleotide substitutions, so that the remaining 14 non-synonymous nucleotide substitutions and three indels narrowed the range of the candidates to four loci, *accD* with 8 amino acid substitutions and 3 indels, *rpoB* with 1 amino acid substitution, *ycf1* with 3 amino acid substitutions, and *ycf2* with 2 amino acid substitutions in the encoded products.

### Search for nuclear counterparts functionally related to plastid candidate loci

A reasonable cause for a nuclear-plastidic conflict is non-complementarity in the structure of polypeptides encoded in the nuclear and plastid genomes which form multisubunit complexes [37]. Therefore, the most plausible plastid candidate locus should have a nuclear-encoded counterpart in the chromosome region known to harbor the *Scs1* gene. The product of *accD*, beta subunit of carboxyltransferase, functions in complex with the alpha subunit of carboxyltransferase, biotin carboxyl carrier protein and biotin carboxylase, together comprising the plastidic heteromeric form of the acetyl-CoA carboxylase [38]. The anticipated counterparts for the *rpoB* gene, which encodes the beta subunit of the so called plastid-encoded RNA polymerase (PEP), are the nuclear-encoded sigma factors, and some other proteins such as CSP41, the iron superoxide dismutase 3, and the plastid transcription kinase PTK (reviewed in [39]). The *ycf1* gene (also named Tic214 in *Arabidopsis*) is involved in preprotein import across the chloroplast inner envelope membrane and supposed to form 1-MD complexes with Tic100, Tic56, and Tic20-I [40]. The *ycf2* is an essential gene of unknown function and its possible nuclear counterparts are unknown [41].

**Fig 1:**
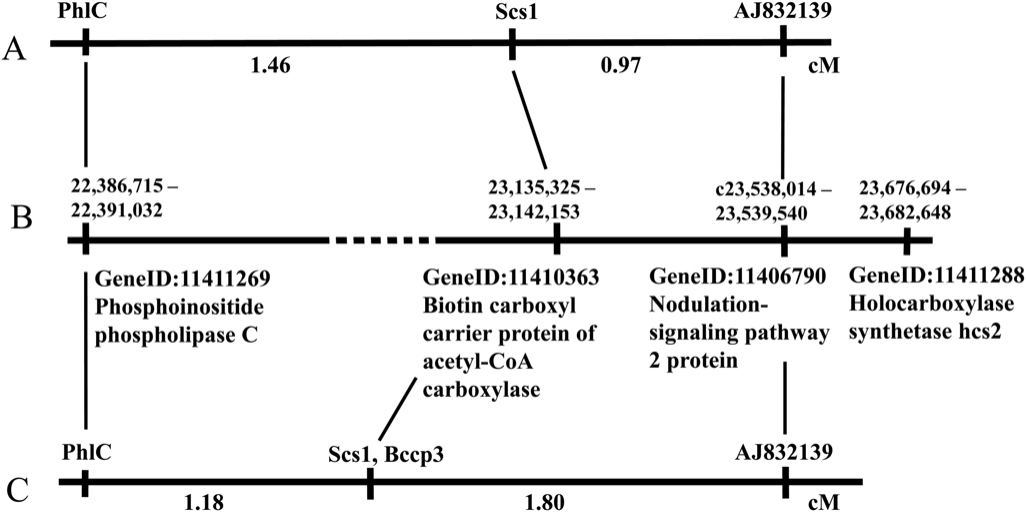
Comparison of the genetic map of pea LGIII and the physical map of *M*. *truncatula* chromosome 3 (NC_016409). A. Pea genetic map based on a cross in the background of the VIR320 cytoplasm (Bogdanova *et al*., 2012). B. Physical map of *M*. *truncatula*. Dashed line stands for a gap of unknown length. C. Pea genetic map based on mapping RIL population in the background of the WL1238 cytoplasm.

The nuclear genome of pea (*Pisum sativum*) is not yet sequenced, therefore, we made use of its synteny with the genome of another legume, *Medicago* [42]. In the annotated *M*. *truncatula* genome, we searched the portion homologous to the pea chromosome region known to harbour the *Scs1* locus for genes encoding subunits of the mentioned plastidic multimeric complexes. The pea *Scs1* was genetically mapped within an interval of about 2.5 cM on Linkage Group III (Fig. 1A) between the loci encoding the phospholipase C and GRAS family protein [22]. The homologs of the bordering markers in *M*. *truncatula* are encoded by the genes referenced as GeneID: 11411269 (Phosphoinositide phospholipase C) and GeneID: 11406790 (Nodulation-signaling pathway 2 protein) which delimit a stretch of DNA sequence between positions 22,386,715 and 23,539,540 of NC_016409 (*Medicago truncatula* chromosome 3), or 1,146,982 bp, of which 150,000 bp refer to a gap of unknown length (Fig. 1B). This region was found to contain 166 annotated genes (S2 Dataset), 70 of which encoded "hypothetical proteins". In the list of the remaining gene products we found the "Biotin carboxyl carrier protein of acetyl-CoA carboxylase", GeneID:11410363 which directly pointed to *accD* as a gene with the complementing function. The position of GeneID:11410363 on the physical map of the *M*. *truncatula* chromosome 3 corresponded well to the position of *Scs1* on the genetic map of pea LGIII (Fig. 1). Among other loci the only gene with relation to plastid function was "Chloroplast lumen common protein family" (GeneID: 11418002) that could hardly be directly regarded as a counterpart of any certain plastid candidate. Thus, we not only substantiated the choice of *accD* as a candidate locus involved in the nuclear-cytoplasmic conflict in the plastid genome, but also nominated the candidate locus for the nuclear counterpart, *Scs1*, on the part of the nuclear genome. Further in the text we designate the pea ortholog of the *M*. *truncatula* GeneID:11410363 as *Bccp3* (according to the *M*.*t*. chromosome).

### Structure of *accD* alleles

**Fig 2:**
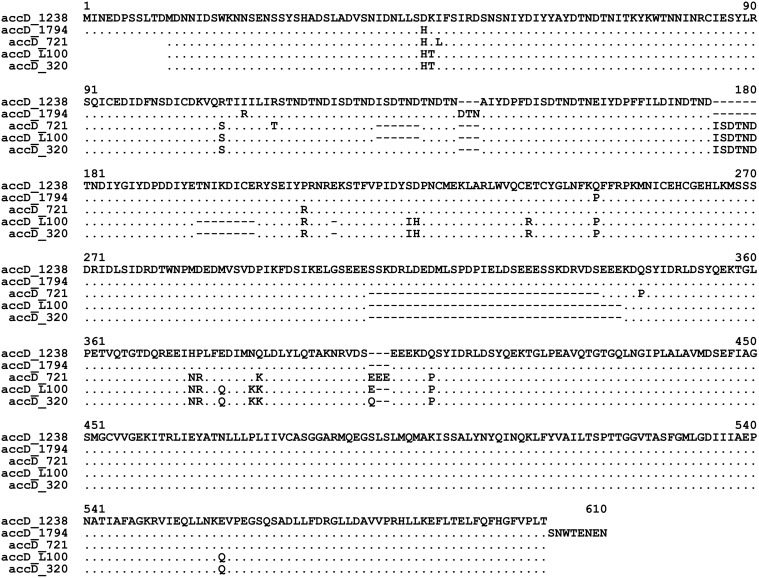
Alignment of the derived amino acid sequences encoded by *accD* alleles of the accessions studied. Dots indicate amino acid residues identical to those of WL1238. Dashes stand for deletions.

The *accD* locus turned to be quite variable. All the studied accessions of wild peas had different structure of the derived protein, while the *accD* allele of WL1238 was identical to that of the reference genome NC_014057 of the cultivar Feltham First, both belonging to the cultivated subspecies *P*. *s*. subsp. *sativum*. Alignment of the derived amino acid sequences encoded by the *accD* alleles from the accessions studied is given in Fig. 2.

The total length of the alignment was 610 amino acids. Apart from 19 variable amino acid positions, there were a number of indels of different size: 1, 3, 6, 8, 31, and 34 amino acid residues, 80 positions in total.

The accessions VIR320, L100 and 721 differed from WL1238 by a 21 bp deletion in the 5' part of the gene that affected both the non-translated region and start codon. Thus, the presumable polypeptide translated from an alternative start codon in VIR320, L100 and 721 had 579 amino acids rather than 590. A mRNA species transcribed from the *accD* of cultivated pea corresponding to a polypeptide of 579 amino acids was observed by Nagano et al. [43]. The accession JI1794 had a nucleotide substitution in the stop codon, so that the C-end of the derived amino acid sequence was 8 amino acids longer than that of WL1238.

The *accD* coding sequence contained numerous short repeats, which might be the cause of indels. The DTN tripeptide repeated many times in the deduced *accD* product in pea as noted yet by Nagano et al. [43] had an additional copy in JI1794 (positions 141–143 in Fig. 2).

The 18-bp stretch ATATCAGATACAAATGAT encoding the hexapeptide ISDTND had two tandemly repeated copies in WL1238, JI1794 and VIR320 (positions numbered 124–135 in Fig. 2), while in 721 and L100 only one copy was present. Besides, the identical copy of this 18-bp sequence was inserted in positions 175–180 (Fig. 2) in VIR320, L100 and 721. Interestingly, an 18-bp motif with one synonymous nucleotide substitution encoding the same hexapeptide ISDTND occured in all accessions studied in the positions numbered 151–156 in Fig. 2.

The differences in the derived amino acid sequences corresponding to the *accD* alleles of the four wild peas as compared with that of WL1238 are summarized in Table 1.

**Table 1:** Variable positions in the alignment of the polypeptidees derived from the *accD* alleles, amino acid changes as compared to WL1238 and their occurrence in the pea accessions studied.

Positions in the alignment	Amino acid change	Present in the indicated accession			
1–11	absence of MINEDPSSLTD (11 AA)		721	L100	320
46	D>H	1794	721	L100	320
**47**	**K>T**			**L100**	**320**
48	I>L		721		
109	R>S		721	L100	320
112	I>R	1794			
116	R>T		721		
130–135	del ISDTND (6 AA)		721	L100	
141–143	ins DTN (3 AA)	1794			
175–180	ins ISDTND (6 AA)		721	L100	320
***196–203***	***del TNIKDICE (8 AA)***			***L100***	***320***
210	P>R		721	L100	320
***214***	***del E***			***L100***	***320***
***224–225***	***SD>IH***			***L100***	***320***
***240***	***E>R***			***L100***	***320***
249	Q>P	1794		L100	320
309–339	del 31 AA		721	L100	320
***340–342***	***del EEE (3 AA)***			***L100***	***320***
345	Q>P		721		
375–376	HP>NR		721	L100	320
***379***	***E>Q***			***L100***	***320***
***383***	***N>K***			***L100***	***320***
384	Q>K		721	L100	320
399	ins E		721	L100	
399	ins Q				320
400–401	ins EE (2 AA)		721		
407	Q>P		721		
***559***	***E>Q***			***L100***	***320***
603–610	addition of SNWTENEN (8 AA)	1794			

Differences occurring in two incompatible cytoplasms, VIR320 and L100, are marked boldfaced and italicised.

In sum, peculiarities of the polypeptide structure in VIR320 and L100, which might be responsible for their incompatibility with *Scs1* of WL1238, were 7 amino acid replacements and three deletions of 8, 1, 3 amino acids (Fig. 2, Table 1).

### Map position of the *Bccp3* locus

The nominated candidate nuclear locus encodes the biotin carboxyl carrier protein (BCCP) of acetyl-CoA carboxylase, its amino acid sequence in *M*. *truncatula* is represented by the XP_003601001 accession. This accession was used as a query for a tblastn search for its pea ortholog in the database of pea Transcriptome Shotgun Assembly (TSA) at NCBI (blast.ncbi.nlm.nih.gov). The pea GAMJ01025269 transcript encoded the amino acid sequence with significant similarity to XP_003601001, 79% identity excluding gaps. The sequence of GAMJ01025269 was used to design primers which were used to amplify and sequence the *Bccp3* locus-specific cDNA from the five pea accessions studied.

The genome of *M*. *truncatula* contains 7 loci annotated as "Biotin carboxyl carrier protein" one of which rather resembles a pseudogene and has no homologies in the available plant databases. To assure that we have chosen the desired locus, we genetically mapped the *Bccp3* using a mapping RIL population obtained as F6 of the cross WL1238 x VIR320. In our earlier studies [22, 23], the allelic state of the loci encoding phospholipase C (*PhlC*) and GRAS family protein (*AJ832139*) was determined via CAPS-analysis and allelic state of the *Scs1* was determined by phenotyping progenies from the crosses of the plants from the mapping population with a testerline. We determined the allelic state of the *Bccp3* locus in 88 RILs and found that this locus was tightly linked to the mentioned LGIII markers. Five RILs were crossovers between *PhlC* and *AJ832139*. In three crossovers, the allelic state of *Bccp3* coincided with that of *PhlC* and in two—with that of *AJ832139* indicating the map position of *Bccp3* between the mentioned markers. In all 88 RILs of the mapping population, the allelic state of the *Bccp3* locus coincided with that of *Scs1* determined by phenotypic analysis, supporting the presumable identity of these loci. A genetic map of the LGIII fragment containing the loci studied was constructed using the Mapmaker 3.0 software as shown in Fig. 1C.

### Structure of *Bccp3* alleles

**Fig 3:**
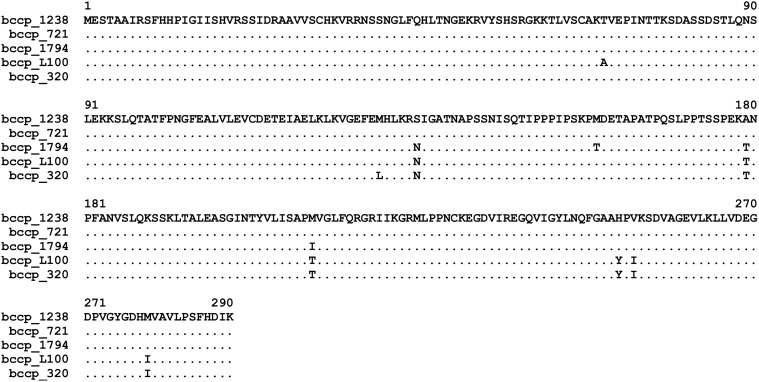
Alignment of the derived amino acid sequences encoded by *Bccp3* alleles of the accessions studied. Dots indicate amino acid residues identical to those of WL1238.

Alignment of the derived amino acid sequences encoded by the *Bccp3* alleles from the accessions studied is given in Fig. 3. Nine variable positions and no indels were recorded, so that all deduced polypeptides had the same length of 290 amino acids. The coding sequence of the WL1238 allele was identical to that of GAMJ01025269, that of the 721 allele differed from them by two synonymous nucleotide substitutions resulting in the same deduced amino acid sequence. Differences in the structure of the Bccp3 allelic variants are summarized in Table 2.

**Table 2:** Variable positions in the alignment of the polypeptides derived from the *Bccp3* alleles, amino acid changes as compared to WL1238 and their occurrence in the pea accessions studied.

Positions in the alignment	Amino acid change	Present in the indicated accession		
70	T>A		L100	
130	M>L			320
135	S>N	1794	L100	320
159	M>T	1794		
179	A>T	1794	L100	320
211	M>I	1794		
***211***	***M>T***		***L100***	***320***
***252***	***H>Y***		***L100***	***320***
***254***	***V>I***		***L100***	***320***
***279***	***M>I***		***L100***	***320***

Differences occurring in two accessions, VIR320 and L100 with incompatible cytoplasms are boldfaced and italicised.

In the preceding section we summarized alterations of the molecular structure characteristic of the *accD* alleles from the cytoplasms incompatible with WL1238 nuclear *Scs1*, namely VIR320 and L100. The cytoplasms of these two accessions are compatible only with the nuclear genomes from the same two accessions, therefore, possible molecular determinants for the interaction of beta subunit of carboxyl transferase encoded by *accD* with biotin carboxyl carrier protein encoded in *Bccp3* are those which distinguish the accessions VIR320 and L100 on one hand from WL1238, JI1794 and 721 on the other hand. There are four such amino acid substitutions in total (Fig. 3, Table 2), all of them confined to the annotated biotinyl domain in the C-part, responsible for biotin binding and present in all biotin-dependent enzymes.

## Discussion

### The nominated candidates in the genetic context

We nominated the candidate loci involved in the nuclear-cytoplasmic incompatibility in pea, *accD* coding for the acetyl-CoA carboxylase beta subunit on the part of the plastid genome, and *Bccp3* coding for the biotin carboxyl carrier protein of acetyl-CoA carboxylase on the part of the nuclear genome. We suppose *Bccp3* to coincide with the earlier described *Scs1* locus responsible for nuclear-cytoplasmic incompatibility [23]. Unlike earlier described cases of nuclear-plastid incompatibility in Solanacea [16] and *Oenothera* [19], here we suppose an impaired protein-protein interaction rather than regulatory incongruence.

The *accD* with *Bccp3* form an ideal pair of candidates as supported by our previous genetic studies [22, 24]. The acetyl-CoA carboxylase (ACCase) is not involved in photosynthesis but plays a key role in biosynthesis of fatty acids, which takes place in plastids [38]. This corresponds well to abnormalities of non-photosynthetic organs such as roots, observed in the plants with the nuclear-cytoplasmic conflict [44].

The plastidic form of ACCase is a multisubunit complex comprised by the biotin carboxylase (BC), biotin carboxyl carrier protein (BCCP) and alpha and beta subunits of carboxyltransferase (alpha-, beta-CT) [38]. The former three subunits are encoded by the nuclear gene, while the latter is plastid-encoded by the *accD* [45].

We suppose that the conflict arises from improper interaction of the subunits encoded in different cellular compartments and co-adapted to bind somewhat evolutionary diverged polypeptides. This situation might be compared with a case of propionic acidemia in humans, a disease caused by dysfunction of the biotin-dependent enzyme propionyl-CoA carboxylase structurally similar to ACCase but consisting of two subunits, alpha and beta [46]. A specific class of mutations resulting in propionic acidemia are those located at the inter—subunit interface which affect multimer formation (reviewed in [47]).

Taking into account the stoichiometry of the holoenzyme (BC)_2_(BCCP)_4_(CT-alpha, CT-beta)_2_ [46] and existence of tissue-specific variants [48], a considerable number of nuclear genes participate in the ACCase formation. It is possible that the nuclear locus *Scs2* [23] encodes one of the subunits of the ACCase complex, thus making *Scs2* complementary to *Scs1*. It is probable that *Scs2* encodes alpha-CT, that is, according to [38], represents the *AccA* locus. The place of *AccA* in the assembly of *M*. *truncatula* and *Cicer arietinum* genomes is unknown, neither it is mapped in pea. We made preliminary mapping experiments and found out that map position of the pea *AccA* is between *Met2* (orthologous to *M*.*t*. Gene ID: 11405476) and *gp*, that is, exactly in the same interval on LGV which harbours *Scs2* [22]. Further experiments are required to determine whether *AccA* and *Scs2* are separable by crossing-over. The multimeric nature of ACCase may also explain the lethality of homozygotes for *Scs2* allele from WL1238 in the background of the VIR320 cytoplasm as depending on segregation of some other nuclear modifier gene(s) [22] which may also encode ACCase subunit(s). One of the *M*. *truncatula* genes annotated as "Biotin carboxyl carrier protein of acetyl-CoA carboxylase", Gene ID: 11442359, is located on chromosome 7 and its pea ortholog was designated by us as *Bccp71*. Segregation of *Bccp71* alleles was found to be distorted in a number of incompatible crosses and to depend on the allelic state of *AccA* among other, still unidentified genetic factors. In reciprocal (compatible) crosses, segregation of *Bccp71* alleles followed a regular Mendelian fashion (unpublished). This scheme corresponds well to the concept of epistatic network regulating allelic interactions of hybrid sterility genes [49].

### Acetyl Co-A carboxylase and compatibility relationships among pea accessions

By now, we have focused on the incompatibility of VIR320 and L100 cytoplasms with the nuclear genome of WL1238. However, the pattern of compatibility among pea representatives is more complicated. While the cytoplasms of WL1238 and VIR320 differ in their compatibility with the *Scs1* allele of WL1238, these both accessions coincide in that they have plastids incompatible with the nuclear genome of 721 [24] (see also S1 Table). This means that there is at least one more determinant underlying plastid incompatibility with nuclear genome.

We have at our disposal the data on compatibility of the accessions studied via segregation analysis in reciprocal crosses [24] as well as data on compatibility in a number of crosses, namely, VIR320 x L100, L100 x VIR320, VIR320 x 721, 721 x VIR320, L100 x 721, 721 x L100 which were analyzed as F1s without further segregation analysis (S1 Table). Based on this knowledge we may try to reconstruct which plastid determinants confer compatibility or incompatibility with the nuclear-encoded counterpart.

In *accD*, there exists a number of sites which may serve as a determinant which makes the plastids of VIR320 and L100 incompatible with the nuclear genome of WL1238. These are seven amino acid substitutions and three deletions (Table 1). One of the amino acid substitutions, E→R in position 240 (Fig. 2) occurred within a zinc finger domain CX_2_CX_15_CX_2_C, which is probably essential for catalytic activity rather than complex formation [50]. On the part of the nuclear counterpart, *Bccp3* (or *Scs1*), possible molecular determinants are represented by four amino acid substitutions in the biotinil domain of BCCP. Any of them or their combinations might be responsible for nuclear-cytoplasmic incompatibility.

Another plastid determinant, as mentioned above, confers incompatibility of the WL1238 cytoplasm with the nuclear genome of 721. The respective nuclear determinant cannot be represented by the same *Bccp3* locus since the polypeptides encoded by the WL1238 and 721 alleles are identical. Whether the same plastid determinant confers incompatibility of the VIR320 cytoplasm with the nuclear genome of 721 is unclear, due to the effect imposed by the *accD—Scs1* interaction. The same is applicable to the L100 accession. To make some assumptions, we may take into account the following consideration. The cytoplasm of WL1238 is incompatible with nuclear genomes of L100 and 721. Therefore, the corresponding nuclear-encoded products in L100 and 721 may have something in common that distinguishes them from their counterparts in WL1238, as well as in VIR320 and JI1794, which are compatible with the WL1238 cytoplasm. We suppose that the nuclear-encoded products possess "binding sites" fitted to molecular determinants in the plastid-encoded products and that differences in the nuclear alleles are paralleled by concordant differences in the plastid alleles. In such a case, the candidate loci to participate in the conflict inferred by the 721 and L100 nuclear genes should be similar in plastid genomes of 721 and L100 but different in the other accessions. In the whole plastid genome, the only candidate which possesses these characteristics is the same *accD* locus. In 721 and L100, it has one copy of the hexapeptide ISDTND (position 130–135 in Fig. 2), which is tandemly duplicated in all other accessions studied. Another possible *accD*-encoded determinant is the glutamic acid residue (E) in the position 399 (Fig. 2). The latter forms part of a negatively charged poly-E stretch which is shorter in WL1238, JI1794 and VIR320 (three residues) than in L100 (4 residues) and 721 (6 residues).

**Fig 4:**
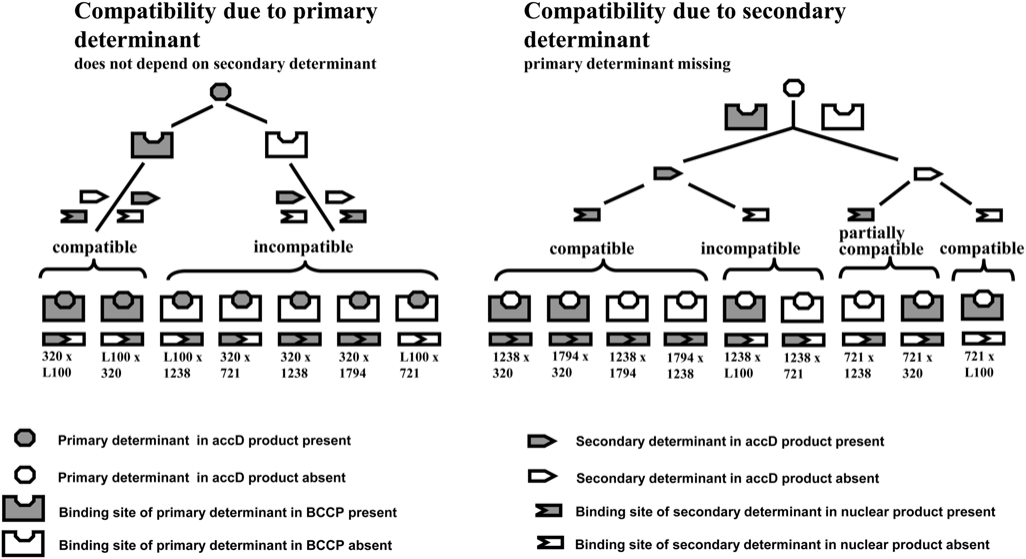
Presumable sheme of compatible and incompatible combinations of molecular determinants in the plastid *accD* and nuclear loci. Partially compatible combinations display decreased pollen fertility but without lethal effects [24].

We suggest the following scheme of compatibility/incompatibility of the determinants encoded by the plastid *accD* and nuclear loci (Fig. 4). We consider a molecular determinant existing in the *accD* product of the accessions VIR320 and L100 to be the primary determinant. If it is present in an *accD* product, such accession is compatible as a donor of cytoplasm only with those accessions where the nuclear-encoded BCCP has the binding site fitted for this determinant (examples: VIR320 x L100, L100 x VIR320), otherwise, the combination is incompatible (examples: VIR320 x WL1238, VIR320 x JI1794, VIR320 x 721, L100 x WL1238, L100 x 721). (Which of the counterparts, nuclear or plastidic, carries "determinant" and which carries "binding site" is conventional and may be equally denominated in the opposite manner.) If an *accD* product lacks the primary determinant, compatibility would depend on the secondary determinant and its respective binding site in some nuclear-encoded product. If the secondary determinant is present in the *accD* product, such cytoplasm would be compatible with the nuclear-encoded product with the fitted binding site (examples: WL1238 x VIR320, JI1794 x VIR320, WL1238 x JI1794, JI1794 x WL1238) and incompatible otherwise (examples: WL1238 x 721, WL1238 x L100). If the secondary determinant is absent from the *accD* product, such cytoplasm would be compatible with nuclear-encoded products, whether they contain the fitted binding site (examples: 721 x VIR320, 721 x WL1238) or not (example: 721 x L100). However, if a nuclear-encoded product contains a vacant binding site for the secondary determinant, such combination would be partially compatible, that is, associated with decreased pollen fertility rather than lethality [24].

Although the presented scheme seems somewhat complicated, it corresponds to actual relationships of compatibility among wild and cultivated peas studied up to now, which are far from being straightforward [22, 24], so that different pea accessions cannot be grouped in such a manner that all members of a group would be compatible within- and incompatible outside the group.

### Nuclear loci involved in the conflict

As for the nuclear-encoded determinants (or binding sites for plastid determinants), we suppose the *Bccp3* locus coding for biotin carboxyl carrier protein to be identical to the earlier described *Scs1* [23] and assume it to carry the primary determinant. Also, we registered lethal effect in the crosses of a number of accessions with WL1238 as donor of the cytoplasm. We believed that this effect was associated with *Scs1* based on the genetic linkage data [24]. According to the present study, it was due to the secondary determinant. However, the here sequenced mRNA of the nuclear *Bccp3* locus cannot encode the primary and secondary determinants at once. Therefore, we have to postulate existence of another locus also linked to the markers of LGIII as shown [24]. We mapped the *Scs1* locus between the markers encoding phospholipase C and GRAS family protein in the cross and RIL population which involved the VIR320 accession [22]. The data on the genotypes of crossover plants observed in the cross involving L100 [21] support this map position. However, if we concern the genotypes of crossover plants registered in the cross involving the accession 721 [24] it turns that they were homozygous for both bordering markers (unpublished) and heterozygous for presumable *Scs1* [24]. This implies either double crossing-over, like that observed in the vicinity of the *scs* locus in wheat [51], or an existence of another closely linked locus outside the interval delimited by phospholipase C and GRAS family protein. Earlier we assumed the "double crossover" version, however, the here presented results rather support existence of another locus. In fact, there is a candidate gene in the chromosome 3 of *M*. *truncatula* at a distance of 534,541 bp from *Bccp3* (that corresponds approximately to 1–2 cM) referenced as Gene ID: 11411288 (Fig. 1B) and encoding "Holocarboxylase synthetase hcs2" containing the "Biotin-(acetyl-CoA carboxylase) ligase" domain assumed to catalyze biotin binding to BCCP. Such a function of the candidate explains its being a secondary determinant as compared to BCCP-encoding gene.

The above considerations open perspective of further investigation of the interaction of the ACCase subunuts via genetic approach.

From the evolutionary point of view, an important role of cytonuclear interaction for establishment of reproductive isolation has been recognized [10, 37]. For example, fertility of barley-wheat alloplasmic lines was found to be associated with heteroplasmy of certain mitochondrial and chloroplast regions [52]. Sometimes a question is raised "Are there particular types of genes that are predisposed to causing hybrid incompatibilities?" [37]. It is tempting to speculate that *accD* might be one of the driving forces of genetic diversification. In various species, *accD* displays high variability, as in *Oenothera* [18], *Medicago truncatula* [53]. The region of the plastid genome where *accD* resides has enhanced variability in legumes [54], or harbours inversion breakpoints associated with complete or partial loss of *accD*, as in Oleaceae [55]. Interestingly, we also registered an inversion breakpoint in the vicinity of *accD* associated with a loss of 29 nucleotides in the intergenic spacer *psaI-accD* in JI1794.

The product of *accD*, beta-subunit of carboxyltransferase, forms a multimeric complex with a number of other subunits. The total number of participants in active complexes is supposed to be ten [46]. All of the nuclear genes coding for proteins potentially interacting with the plastid-encoded *accD* product have to co-adapt to *accD* to compensate for its variability. Such an elevated rate of compensatory amino acid substitution was observed in the sites of nuclear-encoded subunits interacting with mitochondria-encoded components of cytochrome c oxidase complex in primates [56].

In the present work we nominated candidate genes for the involvement in the nuclear-cytoplasmic conflict in the crosses within the genus *Pisum*. Although there still remains a probability of other genes being the cause of the cytonuclear conflict with some non-evident relations among them, the suggested pair of candidates ideally corresponds to the observed patterns of genetic relationships of loci affected by the nuclear-plastid incompatibility. The main participant in the plastid genome is most probably *accD* coding for the acetyl-CoA carboxylase beta subunit, and that in the nuclear genome is the gene *Scs1*, presumably encoding the biotin carboxyl carrier protein of the acetyl-CoA carboxylase. Based on the compatibility relationships among pea accessions and the structure of *Bccp3* alleles we hypothesize existence of another nuclear locus (loci) involved in the conflict mediated by *accD*, possibly *hcs2* encoding biotin-(acetyl-CoA carboxylase) ligase. The variability in the *accD* and *Bccp3* fits our knowledge of cross-compatibility in the genus *Pisum*, while all the spectrum of nuclear counterparts is still to be investigated.

## Supporting Information

S1 Dataset The list of differences between the reconstructed plastid genome of cultivated pea line WL1238 and wild pea accessions VIR320, L100, 721 and JI1794. (XLS)

S2 Dataset The list of annotated genes in the stretch of chromosome 3 of the Medicago truncatula genome (NC_016409) between positions 22,386,715 and 23,539,540, from GeneID: 11411269 (Phosphoinositide phospholipase C) to GeneID: 11406790 (Nodulation-signaling pathway 2 protein). (XLS)

S1 Table Pollen fertility in reciprocal F1 hybrids between pea accessions VIR320, L100 and 721 and in these accessions themselves (the main diagonal). The mean percentage ± standard error (%) of viable (‘fertile’) pollen grains among all pollen grains counted in samples of n flowers from N analysed plants are given.
(PDF)
