# Supplementary material for: Nuclear-Cytoplasmic Conflict in Pea (Pisum sativum L.) Is Associated with Nuclear and Plastidic Candidate Genes Encoding Acetyl-CoA Carboxylase Subunits
Source: PLoS One. 2015 Mar 19;10(3):e0119835. doi: 10.1371/journal.pone.0119835 (PMC4366379; doi:10.1371/journal.pone.0119835)
Supplement: S1 Table — The mean percentage ± standard error (%) of viable (‘fertile’) pollen grains among all pollen grains counted in samples of n flowers from N analysed plants are given. (PDF) [file pone.0119835.s003.pdf]

S1 TABLE

FOR THE PAPER

Nuclear-cytoplasmic conflict in pea (*Pisum sativum* L.) is associated with nuclear and plastidic candidate genes encoding Acetyl-CoA carboxylase subunits.

Vera S. Bogdanova[1], Olga O. Zaytseva[1, 2], Anatoliy V. Mglinets[1], Natalia V. Shatskaya[1], Oleg E, Kosterin[1, 2] and Gennadiy V. Vasiliev[1]

[1]Institute of Cytology and Genetics of the Siberian Branch of Russian Academy of Sciences, Acad. Lavrentyev ave. 10, Novosibirsk, 630090. Russia
[2]Novosibirsk State University, Pirogova str. 2, Novosibirsk, 630090. Russia

S1 Table. Pollen fertility in reciprocal F1 hybrids between pea accessions VIR320, L100 and 721 and in these accessions themselves (the main diagonal).
The mean percentage ± standard error (%) of viable (‘fertile’) pollen grains among all pollen grains counted in samples of *n* flowers from *N* analysed plants are given.

Paternal parent<br>Maternal parent	VIR320	L100	721
VIR320	93.57±2.41<br>*n*=6; *N*=4	71.62±1.92<br>*n*=19; *N*=12	6.08±4.61<br>*n*=5; *N*=1
L100	75.14±2.25<br>*n*=8; *N*=6	96.85±0.65<br>*n*=14; *N*=10	42.65±1.87<br>*n*=12; *N*=6
721	71.6±1.59<br>*n*=12; *N*=6	92.36±0.77<br>*n*=14; *N*=7	94.51±2.34<br>*n*=7; *N*=5

Pollen fertility of F1 hybrids is considered as an indirect indicator of compatibility/incompatibility of the cross performed. Fertility of 50% or less is usually associated with incompatible cross combinations

Plants were grown in a greenhouse in hydroponic beds 4x1 m filled with drainage gravel and fed thrice a day by the standard Knop nutrient solution. Acetocarmine stained pollen (Singh, 2003) was analysed under microscope by counting viable (stained cytoplasm) and dead pollen grains, in total 300-700 grains from an open flower or mature flower bud, with the exception of the VIR320×721 hybrids which were weak and usually did not flower. One plant produced nearly-abortive flowers, some of which had scarce pollen which was analysed.
The data are preliminary, *n* and *N* to be increased.

Singh RJ (2003) Plant Cytogenetics. 2nd ed. CRC Press, Boca Raton. p.21
